# Quantum Oscillations of the Energy Loss Rate of Hot Electrons in Graphene at Strong Magnetic Fields

**DOI:** 10.3390/ma16062274

**Published:** 2023-03-12

**Authors:** Margarita Tsaousidou, Shrishail S. Kubakaddi

**Affiliations:** 1 Materials Science Department, University of Patras, 26 504 Patras, Greece; 2 Department of Physics, K.L.E. Technological University, Hubballi 580 031, Karnataka, India

**Keywords:** energy loss rate, graphene, electron–phonon coupling, acoustic phonons, magnetoquantum oscillations

## Abstract

We present a theoretical model for the calculation of the energy loss rate (ELR) of hot electrons in a monolayer graphene due to their coupling with acoustic phonons at high perpendicular magnetic fields. Electrons interact with both transverse acoustic (TA) and longitudinal acoustic (LA) phonons. Numerical simulations of the ELR are performed as a function of the magnetic field, the electron temperature, the electron density, and the Landau level broadening. We find robust oscillations of the ELR as a function of the filling factor ν that originate from the oscillating density of states at the Fermi level. Screening effects on the deformation potential coupling are taken into account, and it is found that they lead to a significant reduction of ELR, especially, at low electron temperatures, $T_{e}$, and high magnetic fields. At temperatures much lower than the Bloch–Grüneisen temperature, the ELR shows a $T_{e}^{4}$ dependence that is related to the unscreened electron interaction with TA acoustic phonons. Finally, our theoretical model is compared with existing experimental results and a very good quantitative agreement is found.

## 1. Introduction

The discovery of graphene [1,2], a two-dimensional plane of carbon atoms in a hexagonal lattice, has sparked a tremendous interest in understanding its unusual fundamental physical properties [3,4]. The energy dispersion of carriers near the Brillouin zone K point in a zero-magnetic field is that of massless Dirac fermions (i.e., quasi-relativistic like) given by $E_{sk}=s\gamma_{0}\left|k\right|$, where s=+1(−1) for conduction (valence) band, k is the 2D wave vector and $\gamma_{0}=\hbar v_{F}$ with $v_{F}$ being the Fermi velocity. It has been reported that graphene exhibits room temperature mobility as high as $2\times 10^{5}$ cm${}^{2}$/Vs [5,6], and this makes graphene a promising candidate for applications in high-speed devices. At high temperatures, the mobility is limited by electron–phonon (*e–ph*) scattering. The electrons in graphene are weakly coupled to acoustic phonons, while the large optical phonon energy (∼200 meV) makes scattering by optical phonons weak at temperatures up to a few hundred Kelvin. The weak thermal contact between electrons and the lattice is responsible for the high intrinsic carrier mobility in graphene. However, mobility is also influenced by elastic scattering, and for this reason, it is not always possible to extract valuable information about the *e–ph* coupling. On the contrary, the cooling of hot electrons is purely due to phonons, and therefore, the energy loss rate (ELR) is a useful tool for understanding the *e–ph* scattering mechanisms.

In a high electric field, electrons are heated appreciably and driven out of equilibrium with the lattice. An important channel for cooling these “hot electrons” is by emission of acoustic and optical phonons at low and high temperatures, respectively. The weak thermal contact between electrons and phonons in graphene slows down the electron cooling rate. This fact plays a significant role in graphene applications such as bolometry, calorimetry, solar cells, infrared, and THz detectors. The study of electron energy relaxation in graphene has been a subject of extensive theoretical [7,8,9,10,11,12,13,14,15] and experimental [16,17,18,19,20,21,22,23,24,25,26,27] work. We note that because of the large optical phonon energy in graphene, the energy loss of the hot carriers at low temperatures of our interest is primarily due to the emission of acoustic phonons. The energy loss rate, $F(T_{e})$, of hot electrons, with temperature $T_{e}$, in graphene via their coupling to acoustic phonons in a zero-magnetic field has been studied theoretically in Refs. [7,8,9,10,11,12]. In these works, the *e–ph* coupling is described by an unscreened deformation potential, and it is found that in the Bloch–Gruneisen regime ($T_{e}\ll \hbar v_{s}k_{F}/k_{B}$, where $v_{s}$ is the sound velocity and $k_{F}$ the Fermi wave vector) $F(T_{e})$ varies as $T_{e}^{4}$ [7,8,9,10] for clean graphene and $T_{e}^{3}$ in the presence of disorder [11,12,14]. In the latter case, the contribution of the impurity-assisted “supercollisions” to the $T_{e}^{3}$ law is emphasized [11,12,23,25,26]. There is a significant amount of experimental work on cooling of hot electrons in graphene both in the absence [15,16,17,18,20,22,23,24,25,26,27,28,29,30] and in the presence of a magnetic field [21,24,31]. Currently, there is no theory for the ELR of hot electrons in graphene when B≠0. Due to the lack of a relevant theoretical framework, Baker et al. [21,24] used the zero-magnetic field theoretical predictions of Kubakaddi [7] in order to explain the dependencies of the ELR on the electron temperature and sheet density. At low $T_{e}$, good agreement was found between theory and experiment. However, a consistent interpretation within a novel theoretical framework applicable to non-zero-magnetic fields is needed, and this is provided here.

In the present work, we develop the theory for the energy loss rate, ELR, of hot electrons due to their scattering with acoustic phonons in monolayer graphene in quantizing magnetic fields. The calculation is made by using Fermi’s golden rule within the electron temperature model (ETM) [32]. According to ETM, the electrons are assumed to be in thermal equilibrium at temperature $T_{e}$ much higher than the lattice temperature $T_{L}$. Their distribution function is the Fermi–Dirac function. The application of a high magnetic field perpendicular to the plane of the monolayer graphene leads to Landau quantization of the electron energy spectrum. This results in oscillations of the ELR as a function of *B*, F(B), which have the same origin as the Shubnikov–de Haas oscillations in the resistivity. The energy relaxation is studied as a function of the electron temperature, the magnetic field, the electron density, and the width of the Landau level. Finally, we show that our theoretical model explains very well the experimental results of the ELR in exfoliated graphene at high *B* [21].

## 2. Theoretical Method

We consider two-dimensional (2D) electrons of wave vector $k=(k_{x},k_{y})$ moving along the xy plane in monolayer graphene (MLG). In the presence of a perpendicular magnetic field B=(0,0,B), Landau level (LL) quantization occurs, and the energy spectrum is

(1) $$E_{n}=s_{n}\hbar \omega_{B}\sqrt{n}$$

where $s_{n}=1$ for an electron when n>0, $s_{n}=-1$ for a hole when n<0, and $s_{n}=0$ for n=0. The n=0 LL is both the bottom of the conduction band and the top of the valence band. The energy $\hbar \omega_{B}$ is given by

(2) $$\hbar \omega_{B}=\frac{\sqrt{2}\hbar v_{F}}{l_{B}}$$

where $l_{B}=\sqrt{\hbar /eB}$ is the magnetic length. Now, $E_{n}$ takes the form

(3) $$E_{n}=s_{n}v_{F}\sqrt{2\hbar eB}\sqrt{n}.$$

The energy eigenstates are [33]

(4) $$\Psi_{nk_{y}}=C_{n}\frac{e^{ik_{y}y}}{\sqrt{L_{y}l_{B}}}\left[\begin{array}{c} s_{n}\phi_{|n|-1}\left(\frac{x+x_{0}}{l_{B}}\right)\\ \phi_{\left|n\right|}\left(\frac{x+x_{0}}{l_{B}}\right) \end{array}\right]$$

where, $C_{n}={\left[\left(1+\delta_{n,0}\right)/2\right]}^{1/2}$, $L_{y}$ is the dimension of the layer along the *y* direction, $x_{0}=k_{y}l_{B}^{2}$, and $\phi_{n}\left(x\right)=i^{n}{\left(2^{n}n!\sqrt{\pi }\right)}^{-1/2}\exp\left(-x^{2}/2\right)H_{n}\left(x\right)$ with $H_{n}\left(x\right)$ being the Hermite polynomials.

We assume that the 2D carriers are in thermal equilibrium at temperature $T_{e}$ much higher than the lattice temperature $T_{L}$. The carriers lose power due to their coupling with 2D acoustic phonons of wave vector q. The average power loss of the hot electrons is written in the form (e.g., Refs. [32,34,35])

(5) $$F=\frac{1}{N_{e}}\sum_{q,s}\hbar \omega_{q,s}{\left(\frac{dN_{q,s}}{dt}\right)}_{e-ph}$$

where $N_{e}$ is the total number of carriers and $\hbar \omega_{q,s}$ is the phonon energy of a phonon with wave vector q in mode *s* (where, s=LA for longitudinal acoustic phonons and s=TA for transverse phonons). Finally, ${\left(dN_{q,s}/dt\right)}_{e-ph}$ is the rate of change of the phonon distribution function due to the e−ph scattering. This is obtained from Fermi’s golden rule, and it is written as [32,34]

(6) $$\begin{array}{c} {\left(\frac{dN_{q,s}}{dt}\right)}_{e-ph}=g_{s}g_{v}\sum_{nk_{y}}\sum_{n^{\prime }k_{y}^{\prime }}\frac{2\pi }{\hbar }{\left|M_{nn^{\prime }}\left(q,s\right)\right|}^{2}\\ \times \{\left(N_{q,s}+1\right)f\left(E_{n^{\prime }k_{y}^{\prime }}\right)\left[1-f\left(E_{nk_{y}}\right)\right]\\ -N_{q,s}f\left(E_{nk_{y}}\right)\left[1-f\left(E_{n^{\prime }k_{y}^{\prime }}\right)\right]\}\\ \times \delta \left(E_{n^{\prime }k_{y}^{\prime }}-E_{nk_{y}}-\hbar \omega_{q,s}\right)\delta_{k_{y}^{\prime },k_{y}+q_{y}} \end{array}$$

where, $g_{s}$ and $g_{v}$ are, respectively, the spin and the valley degeneracies, $|M_{nn^{\prime }}{\left(q,s\right)|}^{2}$ are the squared e−ph matrix elements (the exact expression is given below), $N_{q,s}\left(T\right)={\left[\exp\left(\hbar \omega_{q,s}/k_{B}T\right)-1\right]}^{-1}$ is the Bose-Einstein distribution at temperature *T*, and f(E) is the Fermi–Dirac distribution function. Finally, the δ-function $\delta (E_{n^{\prime }k_{y}^{\prime }}-E_{nk_{y}}-\hbar \omega_{q,s})$ expresses energy conservation and the Kronecker δ-symbol $\delta_{k_{y}^{\prime },k_{y}+q_{y}}$ imposes momentum conservation in the *y*-direction.

By making use of momentum conservation we replace $k_{y}^{\prime }$ with $k_{y}+q_{y}$ in Equation (6). Now, the rate of change of the phonon distribution function is written in the convenient form

(7) $$\begin{array}{c} {\left(\frac{dN_{q,s}}{dt}\right)}_{e-ph}=g_{s}g_{v}\sum_{nn^{\prime }}\sum_{k_{y}}\frac{2\pi }{\hbar }{\left|M_{nn^{\prime }}\left(q,s\right)\right|}^{2}\left[N_{q,s}\left(T_{e}\right)-N_{q,s}\left(T_{L}\right)\right]\\ \times \left[f\left(E_{nk_{y}}\right)-f\left(E_{n^{\prime }k_{y}+q_{y}}\right)\right]\delta \left(E_{n^{\prime }k_{y}+q_{y}}-E_{nk_{y}}-\hbar \omega_{q,s}\right), \end{array}$$

where, in deriving the above equation, we have used the identities

(8) $$N_{q,s}f\left(E\right)\left[1-f\left(E+\hbar \omega_{q,s}\right)\right]=\left(N_{q,s}+1\right)f\left(E+\hbar \omega_{q,s}\right)\left[1-f\left(E\right)\right]$$

and

(9) $$f\left(E+\hbar \omega_{q,s}\right)\left[1-f\left(E\right)\right]=N_{q,s}\left[f\left(E\right)-f\left(E+\hbar \omega_{q,s}\right)\right].$$

We see that in Equation (7) $k_{y}$ appears only in the energies $E_{nk_{y}}$ and $E_{n^{\prime }k_{y}+q_{y}}$. These are randomized with Landau-level broadening. We can take a simple system average by integrating the above equation over $E=E_{nk_{y}}$ and $E^{\prime }=E_{n^{\prime }k_{y}+q_{y}}$ with a weighting factor $p\left(E-E_{n}\right)p\left(E^{\prime }-E_{n^{\prime }}\right)$ where $E_{n}$ and $E_{n^{\prime }}$ are, respectively, the energies of the *n* and $n^{\prime }$ Landau levels given by Equation (1) and $p(E-E_{n})$ is the broadening of the *n* Landau level [36]. Here we assume a Gaussian form for $p(E-E_{n})$ [36,37]

(10) $$p\left(E-E_{n}\right)=\sqrt{\frac{2}{\pi }}\frac{1}{\Gamma_{n}}\exp\left[-2{\left(E-E_{n}\right)}^{2}/\Gamma_{n}^{2}\right],$$

where $\Gamma_{n}$ controls the LL broadening. Finally, the sum over $k_{y}$ in Equation (7) is replaced by the integral

(11) $$\sum_{k_{y}}\to \frac{L_{y}}{2\pi }\int_{0}^{L_{x}/l_{B}^{2}}dk_{y}=\frac{A}{2\pi l_{B}^{2}}$$

where $A=L_{x}L_{y}$ is the area of the graphene layer.

We assume here that acoustic-phonon scattering does not cause inter-Landau-level transitions, and we set $n=n^{\prime }$ in Equation (7). We note that for the purpose of the present study, this is a fairly accurate approximation. The effect of inter-Landau level transitions on the ELR is examined in detail in a subsequent paper [38]. Now, Equation (7) is written in the form

(12) $$\begin{array}{c} {\left(\frac{dN_{q}}{dt}\right)}_{e-ph}=\frac{g_{s}g_{v}A}{\hbar l_{B}^{2}}\sum_{n}{\left|M_{nn}\left(q,s\right)\right|}^{2}I_{nn}\left(\hbar \omega_{q,s}\right)\\ \times [N_{q,s}\left(T_{e}\right)-N_{q,s}\left(T_{L}\right)], \end{array}$$

where

(13) $$\begin{array}{c} I_{nn}\left(\hbar \omega_{q,s}\right)=\int dE\left[f\left(E\right)-f\left(E+\hbar \omega_{q,s}\right)\right]\\ \times p\left(E-E_{n}\right)p\left(E+\hbar \omega_{q,s}-E_{n}\right). \end{array}$$

The squared matrix elements for the *e–ph* interaction are given by [39,40]

(14) $$|M_{nn}{\left(q,s\right)|}^{2}=\frac{\hbar q}{2A\rho v_{s}}{\left|M_{nn}^{Eff}\left(q,s\right)\right|}^{2}$$

where, ρ is the material density. The square of the ’effective’ *e–ph* matrix elements is [39]

(15) $$|M_{n,n}^{Eff}{\left(q,TA\right)|}^{2}=\Xi_{u}^{2}e^{-u}u\frac{{\left[L_{|n|-1}^{1}\left(u\right)\right]}^{2}}{2|n|}$$

for the transverse acoustic mode and

(16) $$|M_{n,n}^{Eff}{\left(q,LA\right)|}^{2}=\frac{\Xi_{d}^{2}}{\epsilon^{2}\left(q\right)}e^{-u}\frac{{\left[L_{\left|n\right|}^{0}\left(u\right)+L_{|n|-1}^{0}\left(u\right)\right]}^{2}}{4}+\Xi_{u}^{2}e^{-u}u\frac{{\left[L_{|n|-1}^{1}\left(u\right)\right]}^{2}}{2|n|}$$

for the longitudinal acoustic mode.

In the above equations, $u=q^{2}l_{B}^{2}/2$, $L_{\left|n\right|}^{a}\left(u\right)$ (with a=0,1) are the Laguerre polynomials, and ϵ(q) is the static dielectric function. Moreover, $\Xi_{d}$ is the deformation potential constant, and $\Xi_{u}$ is the coupling constant arising from the off-diagonal matrix elements of the *e–ph* interaction. The latter is associated with the shear distortion of the graphene lattice and it is not affected by screening. However, screening reduces significantly the strength of the deformation potential *e–ph* coupling. The interplay between the contributions of the screened deformation potential coupling and the unscreened off-diagonal *e–ph* interaction determines the magnitude and the shape of the ELR oscillations.

In the presence of a strong perpendicular magnetic field the screening dielectric function ϵ(q) has been calculated in several texts [33,36,41,42]. It is written as

(17) $$\epsilon \left(q\right)=1+\frac{e^{2}}{2\kappa_{0}\kappa q}\Pi \left(q\right)$$

where κ is the relative dielectric constant of graphene [33] and $\kappa_{0}$ is the permittivity of free space. We note that the value of κ depends on the substrate. Namely, for graphene on SiO${}_{2}$ substrate $\kappa =(1+\kappa_{SiO_{2}})/2=2.5$. Moreover, Π(q) is the polarization function given by [33,36,41,42]

(18) $$\Pi \left(q\right)=\frac{g_{v}g_{s}}{2\pi l_{B}^{2}}\sum_{n}\Delta_{nn}\left(q\right)p\left(E_{F}-E_{n}\right)$$

where,

(19) $$\Delta_{nn}\left(q\right)=C_{n}^{4}e^{-u}{\left[L_{\left|n\right|}^{0}\left(u\right)+L_{|n|-1}^{0}\left(u\right)\right]}^{2}.$$

The Fermi energy $E_{F}$ is related to the electron sheet density $n_{s}$ by the condition of the electron number conservation (see for example Ref. [36])

(20) $$n_{s}=\int dEf\left(E\right)D\left(E_{F}\right),$$

where $D(E_{F})$ is the density of states at the Fermi energy

(21) $$D\left(E_{F}\right)=\frac{g_{v}g_{s}}{2\pi l_{B}^{2}}\sum_{n}p\left(E_{F}-E_{n}\right).$$

We now return to Equation (5) and by using polar coordinates we replace the sum over the phonon states by

(22) $$\sum_{q}\to \frac{A}{{\left(2\pi \right)}^{2}}\int_{0}^{\infty }qdq\int_{0}^{2\pi }d\phi =\frac{A}{2\pi }\int_{0}^{\infty }qdq.$$

Then, by substituting Equations (12) and (14) into Equation (5) we finally obtain the following expression for ELR

(23) $$\begin{array}{c} F=\frac{g_{v}g_{s}\hbar }{n_{s}4\pi l_{B}^{2}\rho }\int_{0}^{\infty }q^{3}dq\sum_{n,s}{\left|M_{nn}^{Eff}\left(q,s\right)\right|}^{2}I_{nn}\left(\hbar \omega_{q,s}\right)\\ \times \left[N_{q,s}\left(T_{e}\right)-N_{q,s}\left(T_{L}\right)\right]. \end{array}$$

## 3. Results and Discussion

We numerically evaluate ELR as a function of *B*, electron density $n_{s}$, and electron temperature $T_{e}$. Our calculations are performed for $T_{e}$ in the range 1 to 100 K where electron scattering by acoustic phonons is the dominant mechanism for ’hot’ electron cooling [21,24]. The values of the material parameters used here are $g_{s}=g_{v}=2$, $v_{F}=10^{6}$ m/s, $\rho =7.6\times 10^{-7}$ Kg/m${}^{2}$, $v_{TA}=13\times 10^{3}$ m/s, and $v_{LA}=21\times 10^{3}$ m/s [39,40]. The relative dielectric constant of graphene is taken to be κ=2.5 [33]. The lattice temperature is set at $T_{L}=0.1$ K. Moreover, concerning the values of $\Xi_{d}$ and $\Xi_{u}$, we should mention that there is a certain degree of uncertainty (e.g., see Ref. [43] and references therein). Namely, for $\Xi_{d}$ the values quoted in the literature span the range of 9–30 eV. However, the values at the lower end of the range refer mainly to unscreened deformation potential *e–ph* coupling. Concerning the reported values of $\Xi_{u}$, these vary from very low, compared to the deformation potential (e.g., 1.5–4.5 eV [39]) to values comparable to $\Xi_{d}$. Here, we use $\Xi_{d}=30$ eV [44] and $\Xi_{u}=8$ eV. The choice of these values secures good agreement with the experimental data for $F(T_{e})$ in graphene at high filling factors [24] without any other adjustable parameter [38]. In the calculations of the ELR as a function of *B*, the sheet density is taken to be $n_{s}=10^{16}$ m${}^{-2}$. Finally, for the LL broadening, we assume $\Gamma_{n}=\gamma \sqrt{B}$ [39,40] with γ=2 meV/T${}^{1/2}$ unless otherwise specified.

**Figure 1:**
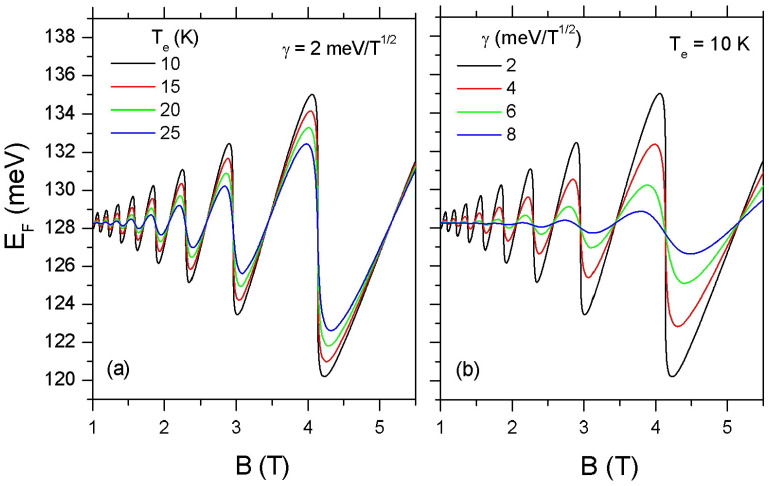
Oscillations of the Fermi energy, $E_{F}$, as a function of magnetic field in an MLG with sheet density $n_{s}=10^{16}$ m${}^{-2}$. In (**a**) the LL broadening parameter γ is taken to be 2 meV/T${}^{1/2}$ and $T_{e}$ varies between 10–25 K while (**b**) depicts $E_{F}$ for γ=2,4,6, and 8 meV/T${}^{1/2}$ at $T_{e}=10$ K.

In Figure 1, we show the numerical estimations of $E_{F}$ as a function of *B* by using Equation (20). $E_{F}$ exhibits sharp discontinuities at half-filling (namely, at ν=4,8,12,16,⋯) which correspond to the nodes of $D(E_{F})$ (see, for example, Ref. [33]). In Figure 1a, we plot $E_{F}$ as a function of *B* at $T_{e}=10,15,20$ and 25 K. The increase of $T_{e}$ weakens the sharp features of the $E_{F}$ oscillations due to the thermal broadening of the Fermi distribution function. In Figure 1b, we see a similar effect on the structure of $E_{F}$ due to the LL broadening.

**Figure 2:**
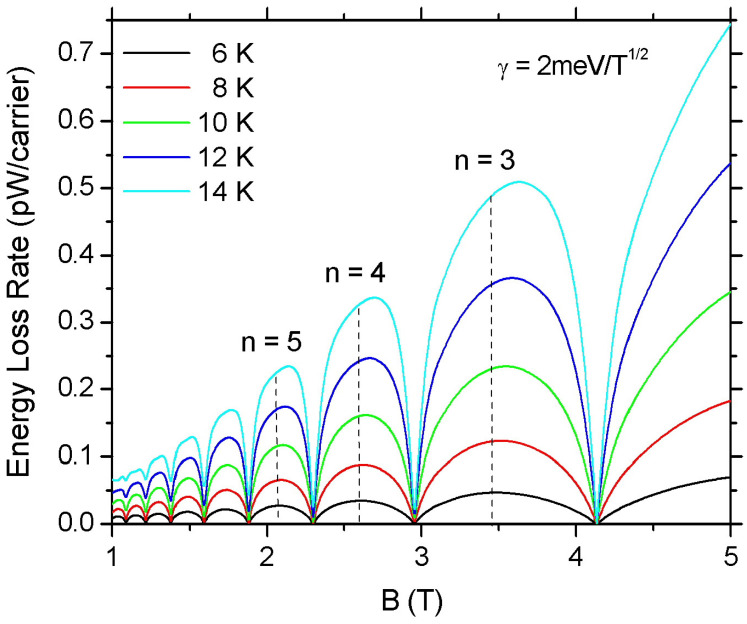
Magnetoscillations of ELR in graphene as a function of the magnetic field for electron temperatures $T_{e}=6,8,10,12$ and 14 K. The electron density is $n_{s}=10^{16}$ m${}^{-2}$ and the LL broadening parameter is taken to be γ=2 meV/T${}^{1/2}$. The positions of the 3rd, 4th, and 5th LL are denoted by the dashed vertical lines.

Figure 2 presents the theoretical values of ELR, F(B), as a function of *B* for $T_{e}=6,8,10,12$, and 14 K. The ELR is calculated by inserting in Equation (23) the Equations (13), (15) and (16). We find that F(B) shows an oscillatory behavior in accordance with the oscillations of the density of states at the Fermi level. Similar behavior for F(B) was predicted previously in GaAs quantum wells [45] and more recently in two-dimensional transition-metal dichalcogenides [46]. The peak values of F(B) occur when $E_{F}$ lies close to the localized state of a LL and appear for values of ν in the vicinity of ν=4(n+1/2) (with n=0,1,2,3,⋯). The nodes of the oscillations correspond to the minima of $D(E_{F})$. The magnitude of F(B) increases with the increase in temperature since more acoustic phonons and more final states become available for *e–ph* scattering. An interesting feature of the F(B) oscillations is the asymmetry observed in the oscillation peaks. This is related to the effect of screening.

**Figure 3:**
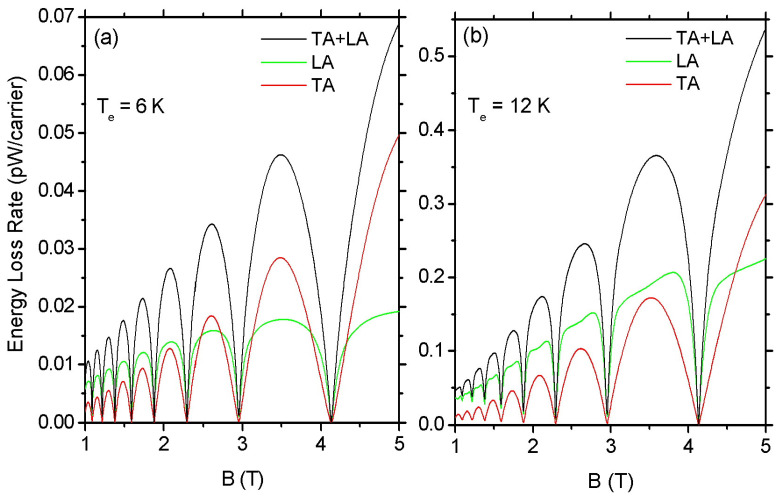
Contribution of TA and LA phonons to the ELR oscillations as a function of *B* at $T_{e}$ = 6 K (**a**) and 12 K (**b**). The black solid line is the total contribution from both TA and LA phonons. The green and red solid lines show, respectively, the contributions of the LA [see Equation (16)] and TA phonons [see Equation (15)]. The contribution of TA overwhelms that of LA phonons at low $T_{e}$ and high *B* due to the strong suppression of the latter by screening. ($n_{s}=10^{16}$ m${}^{-2}$ and γ=2 meV/T${}^{1/2}$).

The shape of the F(B) oscillations is determined by the interplay between the contributions of the screened deformation potential and the off-diagonal *e–ph* coupling. Namely, in Figure 3, the red solid line is the contribution to F(B) arising from the electron interaction with TA phonons, while the green solid line is the corresponding contribution from LA phonons which is mainly due to the screened deformation potential coupling. At low $T_{e}$ and high *B*, the contribution of the TA phonons becomes dominant because screening effects become severe, and they weaken substantially the contribution from the deformation potential coupling. We note that the shape of the F(B) oscillations when the e–ph coupling is determined by screened deformation potential [see Figure 3b] resembles that of magnetothermopower oscillations in 2D electron GaAs/AlGaAs quantum wells [47,48].

**Figure 4:**
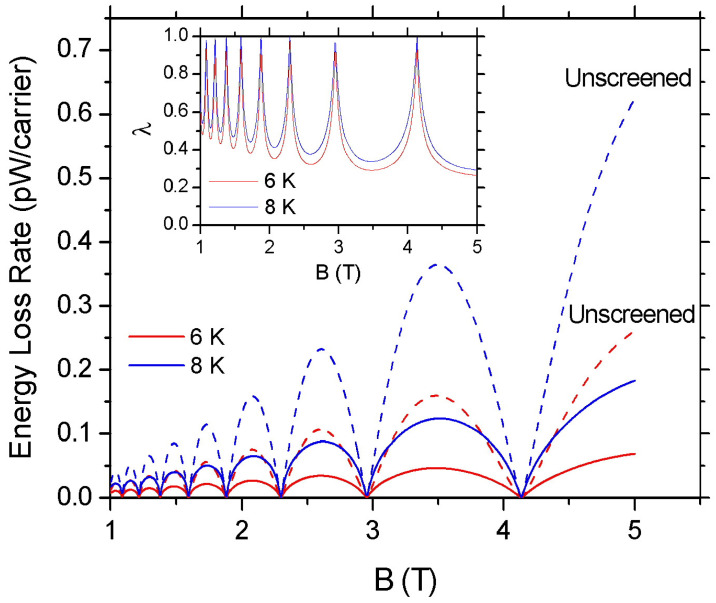
Screening effect on the energy loss rate of an MLG with $n_{s}=10^{16}$ m${}^{-2}$. The solid red and blue lines are the calculated ELR values as a function of *B* when screening is taken into account for $T_{e}=6$ K and 8 K, respectively. The dashed lines show the corresponding results when ϵ(q)=1 (without screening). In the inset, the ratio λ of the screened over the unscreened results is shown as a function of *B* for $T_{e}=6$ K and 8 K. (γ=2 meV/T${}^{1/2}$).

The effect of screening of the deformation potential coupling to the magnitude of F(B) is shown in Figure 4 for $T_{e}=6$ and 8 K. The dashed lines refer to the results when screening is ignored [e.g., ϵ(q)=1 in Equation (16)] and the solid lines to the results when the deformation potential coupling is screened. The inset shows the ratio $\lambda =F^{scr}\left(B\right)/F^{un}\left(B\right)$, where $F^{scr}\left(B\right)$ and $F^{un}\left(B\right)$ are, respectively, the ELR values with and without the consideration of screening. We find that for $T_{e}=6$ and 8 K screening reduces the amplitude of the F(B) oscillations by approximately a factor of 2 at the lowest *B* and by a factor of 3 at the highest *B* examined. We also see that the increase in electron temperature reduces the effectiveness of screening. The dependence of screening on *B* and $T_{e}$ can be explained by inspection of Equations (17) and (18). Namely, the polarization function is proportional to the LL degeneracy ${\left(2\pi l_{B}^{2}\right)}^{-1}$ and consequently increases with *B*. Moreover, the 1/q dependence of the Coulomb interaction results in the enhancement of screening strength as temperature decreases. Finally, at the minima of the F(B) oscillations, the effect of screening is negligibly small due to the collapse of the density of states $D(E_{F})$ at these points.

**Figure 5:**
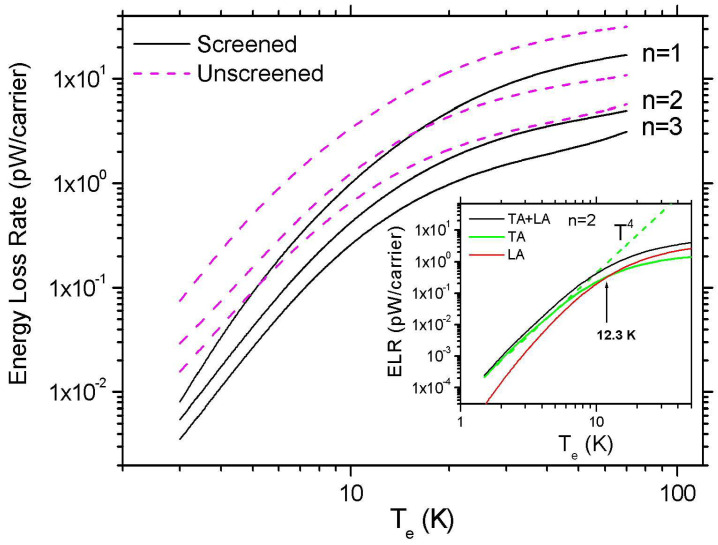
The calculated ELR peaks at n=1,2, and 3 as a function of $T_{e}$. The solid lines are the results when screening is taken into account, and the dashed magenta curves show ELR when screening is ignored. The inset shows the contributions to ELR when n=2 arising from electron coupling with LA (red solid line) and TA phonons (green solid line). At low temperatures, the total ELR follows a $T_{e}^{4}$ law (dashed green line). ($n_{s}=10^{16}$ m${}^{-2}$ and γ=2 meV/T${}^{1/2}$.)

In Figure 5, we evaluate the temperature dependence of the ELR, $F(T_{e})$, when the 1st, 2nd, and 3rd LL becomes occupied with ν=6,10, and 14, respectively. The black solid lines show the calculated $F(T_{e})$ when screening is taken into account, while the dashed magenta lines are the unscreened results. At low temperatures, the ELR shows a $T_{e}^{4}$ behavior. In this case, the magnitude of the ELR is controlled by the off-diagonal *e–ph* matrix elements, which are not affected by screening [40]. Namely, in the inset of Figure 5, the green and the red solid lines show, respectively, the contributions to $F(T_{e})$ from the TA and the LA phonons. (We recall that the contribution of the LA phonons to the *e–ph* is mainly via the screened deformation potential coupling.) The screened deformation potential coupling leads to a much faster decrease in $F(T_{e})$ as temperature decreases, which is described by a $T_{e}^{6}$ law. We should mention that the above $T_{e}$ power laws can be analytically derived from Equation (23) by applying low-temperature approximations that remain valid in the Bloch limit ($q\ll 2k_{F}$) [49].

**Figure 6:**
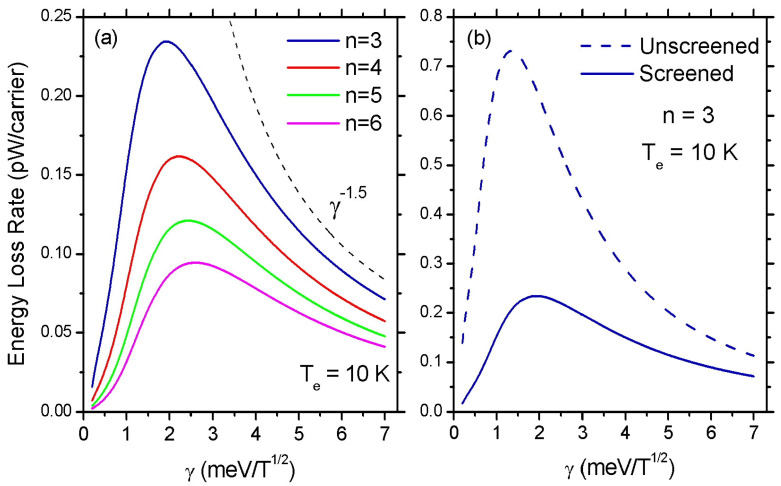
The energy loss rate as a function of γ in an MLG with $n_{s}=10^{16}$ m${}^{-2}$ at $T_{e}=10$ K. In (**a**), the ELR peak values are depicted as a function of the LL broadening parameter when the 3rd, 4th, 5th, and 6th LL is occupied. The black dashed line follows a $\gamma^{-1.5}$ law. In (**b**), the effect of screening on the ELR is shown as γ increases.

So far in our investigation of the ELR, we have assumed a constant value for the LL broadening parameter γ=2 meV/T${}^{1/2}$. Now we will examine how the LL broadening affects the amplitude of the ELR oscillations. In Figure 6a, we show the calculated values of the ELR at n=3,4,5 and 6 as a function of γ at $T_{e}=10$ K. When γ is very small ($\Gamma_{n}\ll k_{B}T_{e}$) the Gaussian weighting factor $p(E-E_{n})$ approaches a δ-function and the intra-LL scattering is strongly suppressed. ELR increases with the increase of γ and reaches a maximum when $\Gamma_{n}\approx \hbar \omega_{q,s}$. When $\Gamma_{n}$ becomes much larger than the phonon energy $\hbar \omega_{q,s}$ the product of the factors $p(E-E_{n})$ and $p(E+\hbar \omega_{q,s}-E_{n})$ becomes proportional to $\gamma^{-2}$. However, due to the occurrence of γ in the polarization function, in Figure 6 we observe a dependence close to $\gamma^{-1.5}$ (black dashed line). We can see from Figure 6a that the peak of ELR moves towards higher values of γ as *B* decreases. A similar shift to a higher γ is observed with the increase of $T_{e}$ (not shown here). In Figure 6b, we show that screening induces a severe suppression of the amplitude of ELR, particularly for small values of γ.

**Figure 7:**
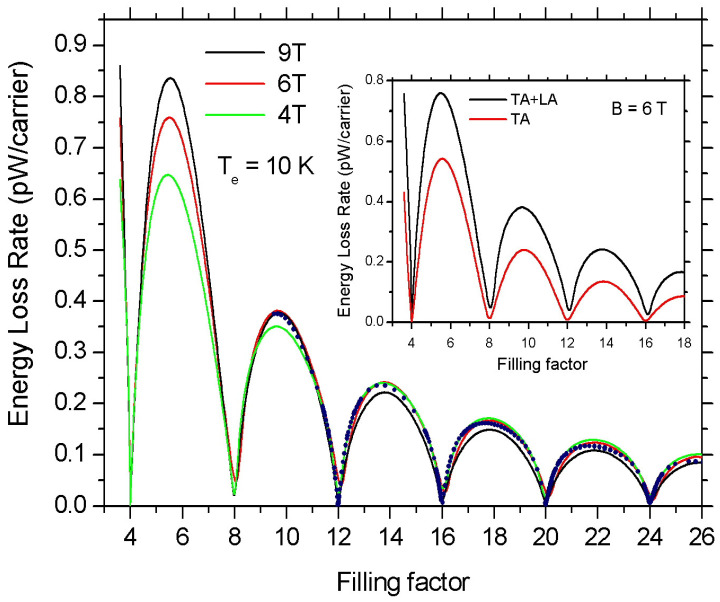
Magnetoscillations of ELR as a function of filling factor, ν, at $T_{e}=10$ K. The green, red, and black solid lines correspond to B=4,6, and 9 T, respectively. Here the ELR oscillations as a function of ν are controlled by the variation of the electron density. For comparison the blue dots show the calculated ELR values when *B* varies between 0.7–5.5 T and $n_{s}=10^{16}$ m${}^{-2}$ at $T_{e}=10$ K. In the inset, the red solid line is the contribution to the total ELR (solid black line) due to electron scattering by TA phonons at B=6 T. (γ=2 meV/T${}^{1/2}$.)

In Figure 7, we show the calculated ELR values as a function of filling factor ν for B=4 T (green solid line), 6 T (red solid line), and 9 T (black solid line) at $T_{e}=10$ K. Here the oscillations of ELR are controlled by the variation of the electron density. We find a remarkable similarity of the oscillations for the three different values of *B*. The inset shows the contribution of the off-diagonal *e–ph* coupling to ELR (red line). Now, in order to compare the two different mechanisms that switch on the oscillations of the ELR (i.e., the variation of *B* when $n_{s}$ is kept constant and the variation of $n_{s}$ at constant *B*) in Figure 7 we present the calculations shown in Figure 2 for $T_{e}=10$ K as a function of filling factor. Namely, the blue dots represent the calculated ELR as a function of ν when $n_{s}=10^{16}$ m${}^{-2}$ and *B* varies between 0.7 and 5.5 T. Once more, a striking similarity is observed.

**Figure 8:**
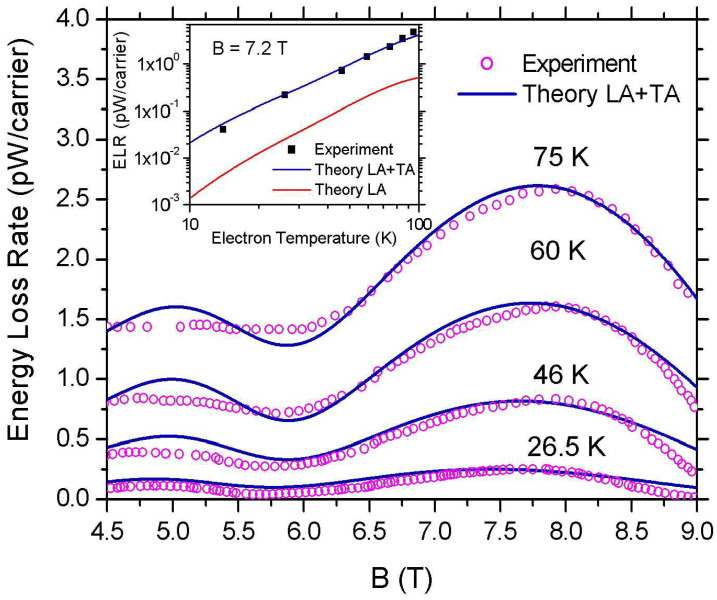
Comparison between theoretical and experimental values [21] of ELR in graphene around ν=6 and ν=10 at electron temperatures in the range 10–100 K. The carrier density is $n_{s}=13.9\times 10^{15}$ m${}^{-2}$. The solid blue lines and the open circles represent, respectively, the theoretical calculations and the experimental data of F(B) obtained as it is explained in the text. In the inset, the filled squares are the experimental data [21] of ELR as a function of $T_{e}$ at ν=6. The solid blue line is the theoretical estimation of $F(T_{e})$ when both LA and TA acoustic modes are considered. For comparison, we show also the contribution from the LA branch when only screened deformation potential coupling is taken into account (red solid line).

In what follows, we compare our theoretical model with relevant experimental data [21] in exfoliated graphene onto a silicon wafer with a 300 nm SiO${}_{2}$ layer. The experimental values of ELR are extracted from the Shubnikov–de Hass (SdH) oscillations of the diagonal resistance $R_{xx}$ as a function of *B* for values of current *I* varying between 1 to 200 μA (e.g., see Figure 2 in Ref. [21]) by using the expression [24]

(24) $$ELR=\frac{I^{2}R_{xx}}{n_{s}A},$$

where $A=5.9\times 10^{-11}$ m${}^{2}$ is the device area and $n_{s}=13.9\times 10^{15}$ m${}^{-2}$. The carrier temperature $T_{e}$ as a function of the input current *I* was obtained from the damped amplitudes of the SdH oscillations. Full experimental details are given in the original papers by Baker et al. [21,24]. The experimental values of ELR as a function of *B* are shown as open circles in Figure 8 for $T_{e}=75,60,46,$ and 26.5 K (top to bottom). In the inset, the filled squares are the experimental data for ELR as a function of $T_{e}$ at ν=6. The lattice temperature in Ref. [21] is $T_{L}=1.5$ K.

The blue solid lines in Figure 8 are the theoretical estimations of the ELR as a function of *B* obtained from Equation (23) and Equations (13), (15) and (16). In these calculations, in order to obtain good agreement with the experiment we take into account the inter-LL and interband contributions in the static dielectric function ϵ(q) [50,51,52]. For simplicity reasons, these contributions were not discussed in our previous analysis. The theoretical results (top to bottom) are obtained for γ=4.2,5.7,7.8, and 9.7 meV/T${}^{1/2}$. We note that the values of the inverse of γ, obtained from the comparison of our theoretical results with the experimental data, show an exponential decay as a function of the inverse carrier temperature. Namely,

(25) $$\gamma^{-1}=\gamma_{c}^{-1}+C\exp\left(-T_{A}/T_{e}\right),$$

where, $\gamma_{c}=9.9\pm 0.2$ meV/T${}^{1/2}$, C=1.7±0.2 meV${}^{-1}$ T${}^{1/2}$, and $T_{A}=190\pm 8$ K. Although the above expression gives a very good quantitative interpretation of the experiment, its origin is not fully understood at the moment.

Now, by using Equation (25) we have calculated $F(T_{e})$ at ν=6 for temperatures in the range of 10–100 K (solid blue line in the inset of Figure 8). As we can see, the agreement with the experimental values (filled squares) is remarkably good. The red line shows the calculated $F(T_{e})$ when only the deformation potential coupling of carriers with LA phonons is considered. Its contribution to the ELR is substantially smaller than the contribution from the off-diagonal e–ph matrix elements at all temperatures examined in agreement with Refs. [39,40]. This is due to the strong suppression of the deformation potential e–ph interaction due to screening. We note that the incorporation of the inter-LL and interband contributions into the dielectric function enhances the screening effect. This is the reason why we do not observe here the crossover between the TA and the LA contributions that we found before (e.g., see inset of Figure 5).

The quantitative interpretation of the experimental data of ELR at low filling factors strongly supports the consistency of our theoretical model. However, in order to explain available experimental data at higher ν (see, for example, Ref. [24]), an extension of the theory is needed in order to include inter-LL e–ph scattering. A detailed theoretical investigation of this scattering mechanism and comparison with the experiment [24] for ν=34,38 and 42 is currently in progress [38].

## 4. Conclusions

We have calculated the rate of energy dissipation (ELR) of ’hot’ electrons in graphene in the presence of a strong perpendicular magnetic field. Electrons cool down through their coupling to acoustic phonons. Our calculations are based on Fermi’s golden rule within the electron temperature model [32]. The calculations are made in a wide electron temperature range of 1–100 K and filling factors 4 to 26. We found pronounced quantum oscillations of ELR as a function of the magnetic field that are related to the oscillations of the density of states at the Fermi level. In order to describe the electron–phonon coupling, we consider both the diagonal (deformation potential) and the off-diagonal e–ph matrix elements by following the analysis of Greenaway et al. [40]. The latter makes a substantial contribution to ELR because of the strong suppression of the deformation potential contribution due to screening effects, especially at low $T_{e}$ and high *B*. At low $T_{e}$, ELR shows a $T_{e}^{4}$ dependence that is related to the dominance of the off-diagonal e–ph coupling. An interesting outcome of our work is the prediction of robust oscillations of ELR as a function of ν that are only slightly affected by the values of the applied magnetic field and the electron sheet density. In addition, we show that our theoretical model explains very well the experimental results of ELR in exfoliated graphene [21] as a function of *B* and $T_{e}$ around ν=6 and 10. In the present study, the inter-LL transitions due to the e–ph scattering have been ignored. Their contribution becomes important at low *B* as $T_{e}$ is elevated. A detailed description of the effect of the inter-LL transitions and comparison with the experiment [24] at large filling factors (ν=34,38, and 42) is given in a subsequent paper [38].

## Data Availability

Data are available from the corresponding author upon reasonable request.
